# Chronic Vitamin D Intoxication in Captive Iberian Lynx (*Lynx pardinus*)

**DOI:** 10.1371/journal.pone.0156331

**Published:** 2016-05-31

**Authors:** Ignacio Lopez, Carmen Pineda, Luis Muñoz, Ana Raya, Guillermo Lopez, Escolástico Aguilera-Tejero

**Affiliations:** 1 Department of Medicina y Cirugia Animal, University of Cordoba, Cordoba, Spain; 2 Hospital Veterinario San Francisco—La Carolina, Jaen, Spain; 3 Proyecto Life Iberlince, Agencia de Medio Ambiente y Agua de Andalucia, Sevilla, Spain; US Geological Survey, UNITED STATES

## Abstract

To document the biochemical and pathologic features of vitamin D intoxication in lynx and to characterize mineral metabolism in healthy lynx, blood samples were obtained from 40 captive lynx that had been receiving excessive (approximately 30 times the recommended dose) vitamin D3 in the diet, and from 29 healthy free ranging lynx. Tissue samples (kidney, stomach, lung, heart and aorta) were collected from 13 captive lynx that died as a result of renal disease and from 3 controls. Vitamin D intoxication resulted in renal failure in most lynx (n = 28), and widespread extraskeletal calcification was most severe in the kidneys and less prominent in cardiovascular tissues. Blood minerals and calciotropic hormones in healthy lynx were similar to values reported in domestic cats except for calcitriol which was higher in healthy lynx. Changes in mineral metabolism after vitamin D intoxication included hypercalcemia (12.0 ± 0.3 mg/dL), hyperphosphatemia (6.3 ± 0.4 mg/dL), increased plasma calcidiol (381.5 ± 28.2 ng/mL) and decreased plasma parathyroid hormone (1.2 ± 0.7 pg/mL). Hypercalcemia and, particularly, hyperphosphatemia were of lower magnitude that what has been previously reported in the course of vitamin D intoxication in other species. However, extraskeletal calcifications were severe. The data suggest that lynx are sensitive to excessive vitamin D and extreme care should be taken when supplementing this vitamin in captive lynx diets.

## Introduction

Vitamin D intoxication has been described in humans [1, 2] and in several species of domestic animals, including cats [3–6]. Free range wild felids should not be affected by vitamin D toxicosis since their carnivorous diet does not contain enough vitamin D to cause clinical problems. However, when maintained in captivity wild felids are often supplemented with vitamins, including vitamin D, and this represents a risk for toxicity.

The Iberian lynx is one of the most endangered species on the planet [7]. This felid was found to be nearly extinct in the early 21^st^ century. During the last decade, a large effort has been dedicated to save this species from extinction, including management of free-ranging populations and implementation of a captive breeding program [8]. Captive Iberian lynx have been reported to suffer a high incidence of chronic renal disease which has been linked to excessive vitamin D supplementation [9].

Excessive vitamin D leads to hypercalcemia and hyperphosphatemia as a consequence of increased intestinal absorption of these minerals and elevated bone resorption [10, 11]. The increased serum calcium x phosphorus product results in soft tissue mineralization and subsequent organ failure [12]. In addition, hypercalcemia has been reported to induce renal failure by reducing kidney perfusion and glomerular filtration [13].

The aim of this study was to document the biochemical and pathologic features of vitamin D intoxication in Iberian lynx and to characterize mineral metabolism in healthy free ranging Iberian lynx.

## Materials and Methods

### Animals

Blood and urine samples obtained from 69 Iberian lynx (*Lynx pardinus*) were used in the study. The population under study included 29 healthy free-ranging lynx (21 males and 8 females, age 3.6 ± 0.4 years, weight 10.8 ± 0.9 kg), and 40 captive lynx that based on their renal function were subsequently classified as healthy captive lynx (4 males and 8 females, age 2.2 ± 0.4 years, weight 9.3 ± 0.4 kg) and lynx with renal disease (16 males and 12 females, age 4.2 ± 0.5 years, weight 10.2 ± 0.4 kg). Renal disease was diagnosed based on 4 criteria: 1) clinical signs (weight loss, poor coat, pale mucous membranes, etc.), 2) azotemia (elevated plasma creatinine and urea concentrations), 3) decreased urine specific gravity (USG), and 4) structural changes in the kidneys detected by ultrasonography and/or radiology. To be classified as having renal disease lynx needed to show azotemia and fulfil at least two additional criteria.

Captive lynx belonged to the *ex situ* Iberian Lynx Captive Breeding Program. These lynx were housed in individual pens with access to a fenced area simulating their natural habitat in the wild. Captive lynx were fed six days/week live prey (rabbit and quail) and meat (rabbit and beef). A nutritional supplement for carnivores fed whole prey (Nutrazu 58QB) provided by Conzoolting Wildlife Management (Barcelona, Spain) was administered 3 times a week. The supplement was labelled to contain 160 IU/kg (dry matter) of vitamin D3 and was administered to provide a dose of approximately 20 IU/kg of body weight. Retrospective analysis (Laboratorio de Diagnostico General, Barcelona, Spain) of 2 samples of supplement showed that they contained 4562 IU/kg and 5460 IU/kg (dry matter) of vitamin D3, respectively. Thus, the lynx were receiving around 600 IU of Vitamin D3/kg of body weight, which is approximately 30 times higher than the recommended dose. The lynx had been receiving this supplement for more than one year.

Handling of the animals was designed to minimize stress. Blood and urine samples were obtained under general anesthesia induced by intramuscular administration of ketamine (5 mg/kg, Ketolar 50 mg/mL, Parke-Davis, El Prat de Llobregat, Spain) and dexmedetomidine (25 μg/kg, Dexdomitor 0.5 mg/mL, Zoetis, Madrid, Spain). Blood samples were drawn from the jugular vein and collected into evacuated tubes containing heparin as an anticoagulant. Samples were centrifuged at 3500 rpm for 10 minutes and plasma was separated. Urine samples were collected by cystocentesis and centrifuged at 1500 rpm for 5 minutes. Both blood and urine samples were obtained from all lynx. Plasma and urine samples were immediately aliquoted and frozen at -20°C until further analysis. Tissue samples (kidney, stomach, lung, heart and aorta) were obtained from 13 captive lynx belonging to the group of lynx renal disease that died as a result of renal failure. Tissue samples were also obtained from 3 free range lynx that that were killed in car accidents. These samples, which were used as controls to compare with the lynx with renal disease, were obtained in the course of a necropsy performed to confirm the cause of death.

Blood and tissue samples were provided by the following institutions: Banco de Germoplasma y Tejidos de Especies Silvestres Amenazadas (Madrid), Banco de Recursos de la Universidad Miguel Hernandez (Elche), Centro de Analisis y Diagnostico de la Fauna Silvestre de Andalucia (Malaga), Centro de Cria en Cautividad La Olivilla (Jaen), Centro de Cria en Cautividad El Acebuche (Huelva) and the LIFE-Lince Project. All protocols, including ethics, were reviewed and approved by the Ex-situ Breeding Program for Iberian Lynx and the Consejeria de Medioambiente y Ordenacion del Territorio—Junta de Andalucia (Spain).

### Blood Chemistries

Plasma calcium, phosphorus, magnesium, urea and creatinine were quantified by spectrophotometry (BioSystems S. A., Spain). Plasma concentrations of 25(OH)-vitamin D (calcidiol) and 1,25(OH)_2_-vitamin D (calcitriol) were measured using radioimmunoassays (Immunodiagnostic Systems Ltd, UK) that have been validated for cats (calcidiol: intra-assay coefficient of variation (CV) 6%, inter-assay CV 8%, sensitivity of the assay <2 ng/mL; calcitriol: intra-assay CV 9%, inter-assay CV 12%, sensitivity of the assay <3 pg/mL) [14]. PTH was measured using an immunoradiometric assay (Scantibodies Laboratory Inc, USA) designed for the quantitative determination of human “intact” PTH and validated for measurement of feline PTH (intra-assay CV 9.5%, inter-assay CV 9.8%, sensitivity of the assay = 2 pg/mL) [15]. All measurements were performed in the same laboratory.

### Urine Chemistries

Calcium, phosphorus, magnesium and creatinine were quantified by spectrophotometry (BioSystems S. A., Spain). USG was measured by refractometry (Zuzi Auxilab SL, Spain). Fractional excretion (FE) was calculated according to the following equation:

$$FE\;(\%)=[\left(U_{e}\times P_{creat})/(P_{e}\times U_{creat}\right)]\times 100,$$

where U_e_ is the urine electrolyte concentrations, P_creat_ is the plasma creatinine concentration, P_e_ plasma electrolyte concentration and U_creat_ is the urine creatinine concentration.

### Assessment of tissue calcification

Fresh tissue was fixed in 10% buffered formalin, embedded in paraffin and cut into 3 μm sections. Samples were not decalcified. Paraffin-embedded sections of kidney, stomach, lung, heart and aorta were stained with hematoxylin-eosin and von Kossa stains. The extent of extraskeletal calcification was calculated by a semiquantitative score obtained from the von Kossa stained samples. Two independent observers, which were blinded to the samples, scored each sample on a scale from 0–3 (0 = no calcification, 1 = slight calcification, 2 = moderate calcification, 3 = severe calcification).

### Statistics

Statistical analysis was performed using the computer package SPSS for Windows 15.0 (SPSS Inc., Chicago, IL, USA). Values are expressed as the mean ± standard error (SE). The difference between means was assessed by one-way ANOVA followed by a LSD post-hoc test. *p*<0.05 was considered significant.

## Results

**Fig 1:**
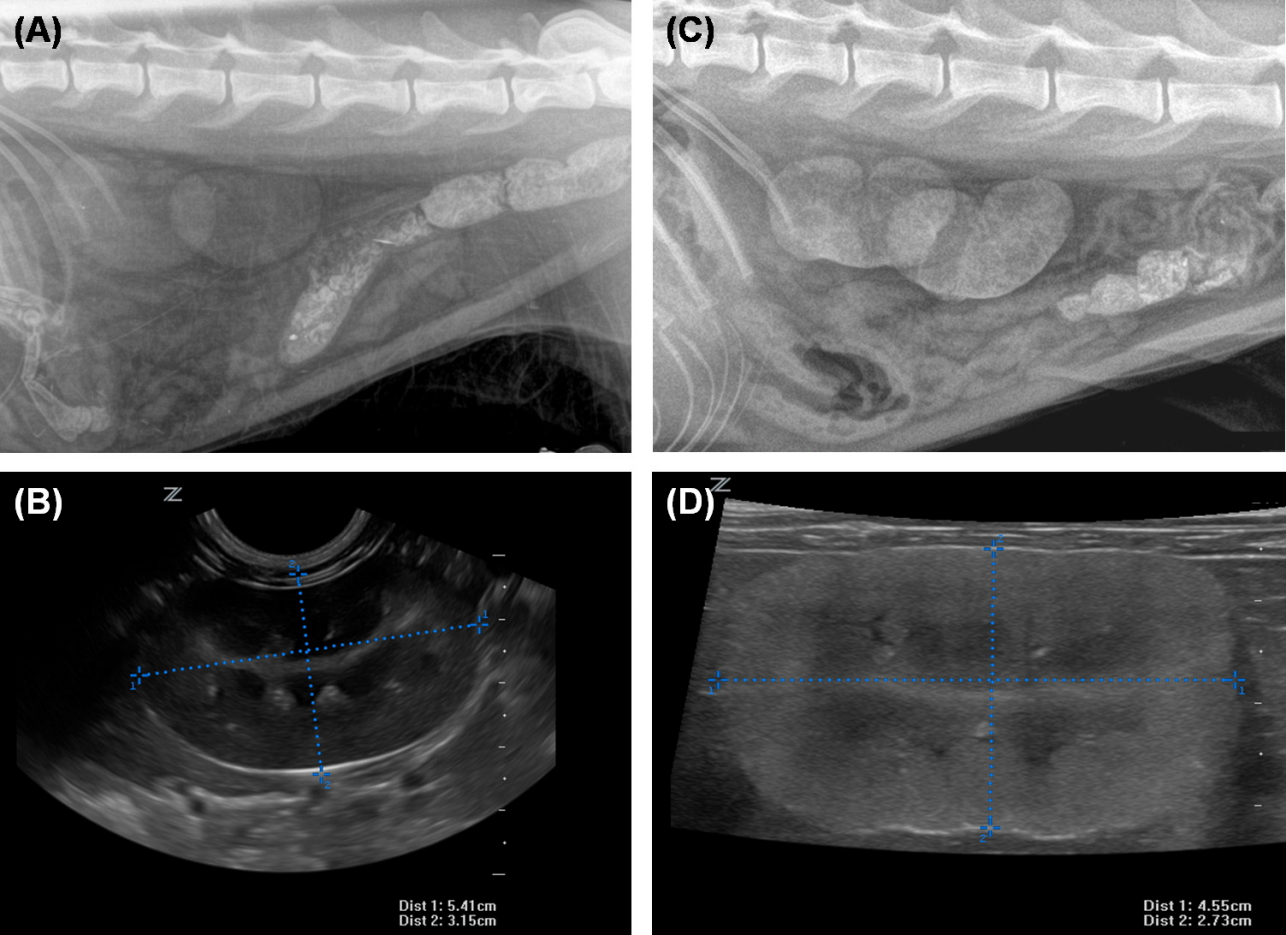
Ultrasonographic and radiologic images of the kidneys. (A, B) healthy lynx, (C, D) lynx with renal disease. Diseased kidneys are smaller, have irregular contour, show loss of normal architecture and mineralization.

Healthy lynx had plasma creatinine and urea concentrations within the reference ranges for the species and no differences were found between free range and captive groups. As expected, lynx with renal disease had increased values of creatinine (4.2 ± 0.4 mg/dL) and urea (161 ± 14.6 mg/dL). Lynx with renal disease had significantly lower USG (1.023 ± 0.0042) than both healthy captive lynx (1.062 ± 0.004) and free range lynx (1.074 ± 0.002). Small and irregular kidneys, showing loss of normal architecture and calcification foci, were identified in most lynx with renal disease by either ultrasonography or radiology (Fig 1).

Plasma calcium was within normal limits in healthy lynx, although captive animals had significantly higher values than free-ranging lynx. By contrast, lynx with renal disease had higher calcium than the other two groups and their values were outside the reference range (12.0 ± 0.3 mg/dL). Plasma phosphorus showed the same trend as calcium although in this case no significant differences were found when comparing free range and healthy captive lynx, and hyperphosphatemia was moderate (6.3 ± 0.4 mg/dL) in lynx with renal disease. No differences were observed in plasma magnesium concentrations in the three groups of lynx. FE of calcium, phosphorus and magnesium were similar in wild-ranging and healthy captive lynx. However, FE of calcium (2.3 ± 0.5%), phosphorus (31.6 ± 2.3%) and magnesium (4.2 ± 0.8%) were significantly elevated in lynx with renal disease. Although plasma PTH concentrations tended to be higher in wild-ranging lynx than in captive lynx, no significant differences were detected between the two groups of healthy lynx. However, lynx with renal disease had very low PTH values (1.2 ± 0.7 pg/mL). Plasma calcidiol concentrations in healthy free range lynx were 74.9 ± 8.9 ng/mL. Captive lynx had higher plasma calcidiol concentrations, and calcidiol was significantly elevated in lynx with renal disease (381.5 ± 28.2 ng/mL), *p*<0.05 vs captive healthy lynx (243.4 ± 69.2 ng/mL). Healthy free-ranging lynx had plasma calcitriol concentrations of 193.5 ± 19.2 pg/mL. Plasma calcitriol concentrations were elevated only in healthy captive lynx (364.4 ± 94.9 pg/mL), *p*<0.05 vs captive lynx with renal disease (212.1 ± 15.6 pg/mL) (Table 1).

**Table 1:** Blood and urine parameters in healthy free ranging lynx, healthy captive lynx and captive lynx with renal disease.

Parameters	Healthy free ranging (n = 29)	Healthy captive (n = 12)	Renal disease captive (n = 28)
Creatinine (mg/dL)	1.26±0.11	1.29±0.11	4.24±0.37^a^^b^
Urea (mg/dL)	77.8±3.38	59.4±1.91	161.12±14.62^a^^b^
Calcium (mg/dL)	8.68±0.26	9.62±0.46^a^	10.96±0.29^a^^b^
Phosphorus (mg/dL)	5.32±0.29	5.97±0.42	6.35±0.36^a^
Magnesium (mg/dL)	2.99±0.29	2.78±0.15	2.46±0.12
Parathyroid hormone (pg/mL)	6.75±1.88	3.77±1.36	1.23±0.69
25 (OH) vitamin D (ng/mL)	74.90±8.88	243.40±69.19^a^	381.52±28.19^a^^b^
1,25 (OH)_2_ vitamin D (pg/mL)	193.54±19.17	364.41±94.88^a^	212.11±15.55^b^
Urine specific gravity	1.074 ± 0.002	1.062 ± 0.004	1.023 ± 0.0042^a^^b^
FE calcium (%)	0.14±0.02	0.07±0.03	2.33±0.47^a^^b^
FE phosphorus (%)	14.43±1.15	17.12±1.87	31.6±2.35^a^^b^
FE magnesium (%)	0.83±0.13	1.25±0.30	4.21±0.80 ^a^^b^

25 (OH) vitamin D, calcidiol; 1,25 (OH)_2_ vitamin D, calcitriol; FE, fractional excretion. Values are expressed as the mean±SE.

^a^*p*<0.05 vs healthy free ranging

^b^*p*<0.05 vs healthy captive.

**Fig 2:**
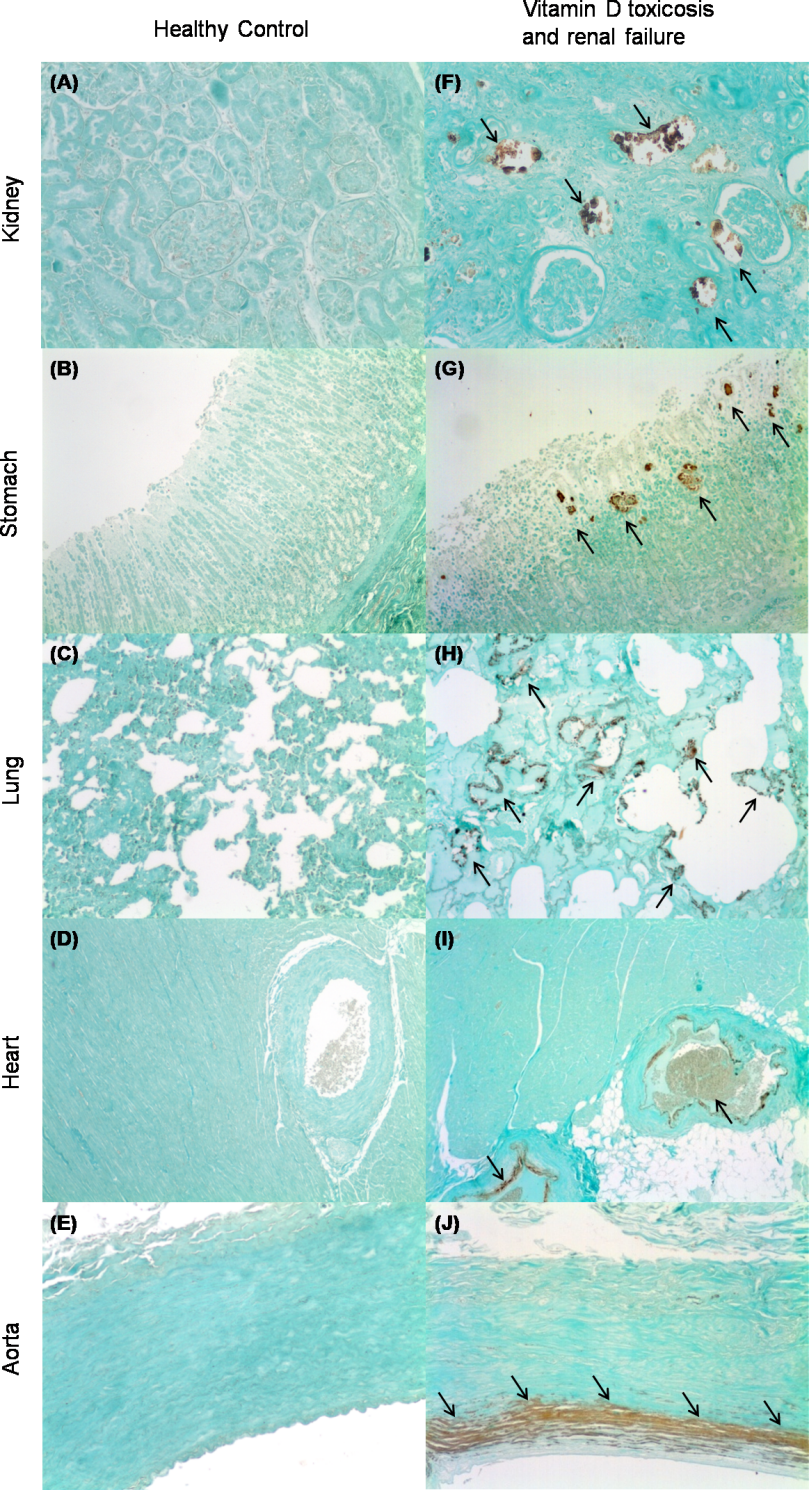
Von Kossa stained sections of the kidney, stomach, lung, heart and aorta from Iberian lynx. (A-E) Healthy controls that died in car accidents; (F-J) Animals that died as a consequence of vitamin D toxicosis and renal failure. Foci of calcification (arrows) are stained brown. Magnification = x200.

Fig 2 shows extraskeletal calcification in captive lynx that died as a consequence of chronic renal disease. When tissue samples were scored for calcification, mineral deposition was more prominent in the kidneys (score = 2.0 ± 0.2). Calcifications were also extensive in stomach (score = 1.1 ± 0.2) and lung (score = 1.2 ± 0.4). In cardiovascular tissue, although focal calcified areas were also observed, the calcification scores were lower with values of 0.5 ± 0.2 in the aorta and 0.3 ± 0.2 in the heart. Renal calcifications were widespread and affected both glomeruli and renal tubules. In the stomach, calcifications were mainly found in the mucosa. Aortic calcification was restricted to the tunica media.

## Discussion

The central objective of this study was to characterize the main features of vitamin D toxicosis in lynx and to gain insight into the associated changes in mineral metabolism. As a wild species, information about normal values of biochemical parameters in lynx is limited. Thus, although the number of samples was not enough to establish reference values, a second objective, which was accomplished by the study of samples from free range lynx, was to provide normal values for parameters of mineral metabolism in healthy lynx.

Plasma calcium and phosphorus concentrations in free range Iberian lynx were in accordance with previously published data [16]. It is important to note that plasma calcium and phosphorus in lynx are similar to domestic cats and this is relevant for comparison of calciotropic hormones, which have been studied in cats but not in lynx. Plasma PTH concentrations in healthy lynx were similar to what has been recently described in cats using the same PTH assay [15]. Calcidiol concentrations in plasma of healthy lynx were similar to values reported in adult cats; however, plasma calcitriol concentrations tended to be higher in lynx than in adult cats [14].

In this study captive lynx were allotted in two groups: healthy lynx and lynx with renal disease. This classification is useful because renal disease was the main clinical feature associated with vitamin D toxicosis; however, the healthy captive lynx were also exposed to excessive vitamin D intake and thus may be viewed as a population of lynx with subclinical vitamin D intoxication.

An increase in extracellular calcium is the main feature of vitamin D toxicity in many species [3–5, 10]. Captive lynx showed elevated plasma calcium concentrations when compared with free range lynx, however the magnitude of hypercalcemia was moderate and much lower than what has been reported in clinical cases of vitamin D toxicosis in domestic cats [6, 17–19]. Previous reports of vitamin D intoxication in cats have mostly described acute toxicosis due to ingestion of vitamin D-containing rodenticides [6, 17, 18], which is different to the prolonged exposure to supplements with high vitamin D concentrations to which captive lynx were subjected. In the lynx with subclinical toxicosis this moderate degree of hypercalcemia was not surprising and the absence of marked hypercalcemia may be explained by the hormonal adjustments after chronic exposure to high doses of vitamin D as backed up by low PTH. In captive lynx with renal disease calcium concentrations were higher but still lower than what has been reported in vitamin D intoxicated cats [6, 17–19]. In these lynx, renal failure may have influenced plasma calcium since cats with renal disease tend to show hypocalcemia [20]. Thus, the hypercalcemia of vitamin D intoxication may have been counterbalanced, to some extent, by the hypocalcemia of renal disease.

Captive lynx had increased plasma phosphorus concentrations and changes in phosphorus paralleled changes in calcium concentration. As with calcium, hyperphosphatemia was less severe than previously reported in vitamin D toxicosis in other species. In this case the moderate hyperphosphatemia found in lynx with renal disease was particularly remarkable, as hyperphosphatemia is one of the biochemical hallmarks of renal disease in cats [20], the effects of vitamin D intoxication and renal disease on plasma phosphate concentrations should be additive. The absence of frank hyperphosphatemia might have been related to the chronic nature of the intoxication or to specific changes in lynx mineral metabolism in the course of renal disease.

Plasma PTH was lower in captive lynx than in the free range population. The decrease in PTH, which has been previously documented in animals with vitamin D toxicosis, is likely secondary to hypercalcemia and to the inhibitory effects of vitamin D metabolites on PTH production by the parathyroid glands [21]. A reduction in serum PTH concentrations is uncharacteristic for feline renal disease—cats with chronic kidney disease are often hypocalcemic and therefore tend to develop secondary hyperparathyroidism and have increased serum PTH [20]. Low PTH and hypercalcemia without hyperphosphatemia have been reported in horses and rabbits with renal failure and the decreased PTH has been attributed to the inhibitory effects of hypercalcemia [22, 23].

Plasma calcidiol concentrations were markedly elevated in captive lynx. Calcidiol is considered the serum biochemical marker that best reflects vitamin D ingestion since it is produced by the liver in direct correlation with dietary intake [24]. Thus, the increase in plasma calcidiol confirms excessive intake of vitamin D.

Plasma calcitriol was elevated in captive healthy lynx but not in lynx with renal disease. In animals with vitamin D intoxication plasma calcitriol has been reported to be decreased [21, 25]. The explanation for decreased calcitriol in the course of vitamin D intoxication is not clear and several mechanisms may be implicated. The activity of the enzyme 1-alpha-hydroxylase (CYP27B1) which is responsible for metabolizing calcidiol into calcitriol is downregulated by increased calcium and decreased PTH, which are features of vitamin D intoxication [24]. Studies in dogs have also shown upregulation of 24-hydroxylase, an enzyme that metabolizes calcitriol, in vitamin D intoxication [21]. The increase in calcitriol found in the captive lynx without renal disease may be related to the moderate hypercalcemia and non-significant decrease in PTH, which would be insufficient to block calcitriol production. In lynx with renal disease, that had more severe hypercalcemia and lower PTH, calcitriol was lower than in healthy captive lynx but was not decreased when compared with free ranging lynx.

Since calcitriol is about 100-fold more able to bind the vitamin D receptor (VDR) than calcidiol, it is also the metabolite with the greatest toxic potential [24]. However, as above discussed, in vitamin D intoxication serum calcitriol concentrations are typically decreased. It has been hypothesized that the increase in serum calcidiol may displace serum calcitriol from vitamin D transporting protein increasing free calcitriol [11, 21, 24], which would be able to bind to VDRs. However, recent studies with CYP27B1 knockouts support the concept that calcidiol rather than calcitriol is responsible for vitamin D toxicity [25]. In the lynx of this study, the effect of the increased calcidiol concentration may have been potentiated by the normal (non-decreased) calcitriol concentrations and this may have contributed to the severity of soft-tissue calcifications.

Vitamin D intoxication leads to extraskeletal calcification and organ dysfunction as a consequence of mineral deposition in soft tissue [17, 26]. Extraosseous mineralization is not entirely a passive process and in the case of blood vessels vascular calcification involves a phenotypic transformation of smooth muscle cells from muscular to osteogenic phenotype [27]. Although vitamin D metabolites may have some direct effect on soft tissue mineralization, this process is thought to be mediated mostly by the increased calcium x phosphorus product as a consequence of hypercalcemia and hyperphosphatemia [12]. An interesting characteristic of the cases reported here is that this product was not markedly increased, due mostly to the small elevation in serum phosphorus concentration. Another remarkable feature is that, when compared with experimental models of uremia-associated extraskeletal calcifications [28], vascular calcification was moderate. Widespread soft tissue mineralization, similar to what we are reporting here, has been recently described in three tigers which died in zoological gardens. Although the etiology was not confirmed, the authors suspected vitamin D overdose [29].

## Conclusions

In Iberian lynx with chronic vitamin D toxicosis the associated changes in mineral metabolism show some differences with what has been previously reported in man and animals (mainly, moderate hypercalcemia and hyperphosphatemia). However, extraskeletal calcifications were severe. The high plasma calcitriol concentrations identified in healthy lynx and the fact that calcitriol did not decrease in lynx with vitamin D intoxication may have influenced the severity of extraosseous calcifications. Soft tissue calcifications were more accentuated in the kidney and less severe in cardiovascular tissue. All these data suggest that lynx are sensitive to excessive vitamin D and extreme care should be taken when supplementing this vitamin in captive lynx diets.
